# Pediatric Atypical Mycobacterium Infection Presenting as Wheezing and Concern for Foreign Body Aspiration

**DOI:** 10.7759/cureus.491

**Published:** 2016-02-12

**Authors:** Nandini Govil, Prasad John Thottam, Darshit J Thakrar, David H Chi

**Affiliations:** 1 Department of Otolaryngology, University of Pittsburgh Medical Center; 2 Michigan Pediatric Ear, Nose and Throat Associates, Children’s Hospital of Michigan, Detroit Medical Center; 3 Department of Pediatric Radiology, Children’s Hospital of UPMC; 4 Department of Pediatric Otolaryngology, Children’s Hospital of UPMC

**Keywords:** mycobacterium avium complex, differential diagnosis, foreign body aspiration, pediatric airway, pediatric otolaryngology

## Abstract

Atypical mycobacterium infection most commonly presents as asymptomatic cervical lymphadenitis in immunocompetent children. Over the last several decades, rates of Mycobacterium avium complex (MAC) infection have been increasing in both number and severity, with more cases of pulmonary infection reported in healthy children. However, guidelines on how to treat children with these infections remain unclear. The presentation of this disease is variable and often presents with an indolent course of wheezing that is misdiagnosed as foreign body aspiration. Several case reports have described successful treatment of these children with surgical excision without the need for additional treatment with antimycobacterial agents. We present the case of a healthy 20-month old male with wheezing and concern for foreign body ingestion. Rigid bronchoscopy demonstrated a left bronchus mass. The patient underwent video-assisted thoracoscopic surgery (VATS) with improvement in respiratory symptoms. Final pathology showed necrotizing granulomatous infection consistent with MAC. This report demonstrates the importance of keeping intrathoracic MAC infection in the differential when evaluating an immunocompetent child with wheezing or shortness of breath.

## Introduction

Nontuberculous mycobacteria (NTM) infection is increasing in incidence and severity in developed countries [1]. One disease-causing NTM in children is Mycobacterium avium complex (MAC), a common environmental pathogen found in soil, water, and house dust [2]. Atypical mycobacterium infection with MAC in childhood usually presents as an isolated, cervical lymphadenitis. Systemic or invasive involvement is rare, usually only occurring in children with T-cell immunodeficiency. With the increasing incidence of MAC infections, reports of more severe and disseminated intrathoracic infection in immunocompetent children have been documented [2-6]. Guidelines on how to treat immunocompetent children with intrathoracic MAC are unavailable; the type and extent of surgical excision offered as well as the role of treatment with antimycobacterial drugs remain unclear. We describe an immunocompetent toddler with a MAC-associated mediastinal mass successfully treated with video-assisted thoracoscopic surgery (VATS) excision.  

## Case presentation

A previously healthy, 20-month male presented to our pediatric hospital with a two-week history of wheezing refractory to albuterol and prednisone. Chest x-ray demonstrated left lung air trapping and left bronchus narrowing concerning for foreign body aspiration. No witnessed ingestion was reported, but the patient had a positive history of playing in soil and putting rocks in his mouth. Upon exam, he had coarse breath sounds bilaterally and wheezing in left lung fields on auscultation.

**Figure 1:**
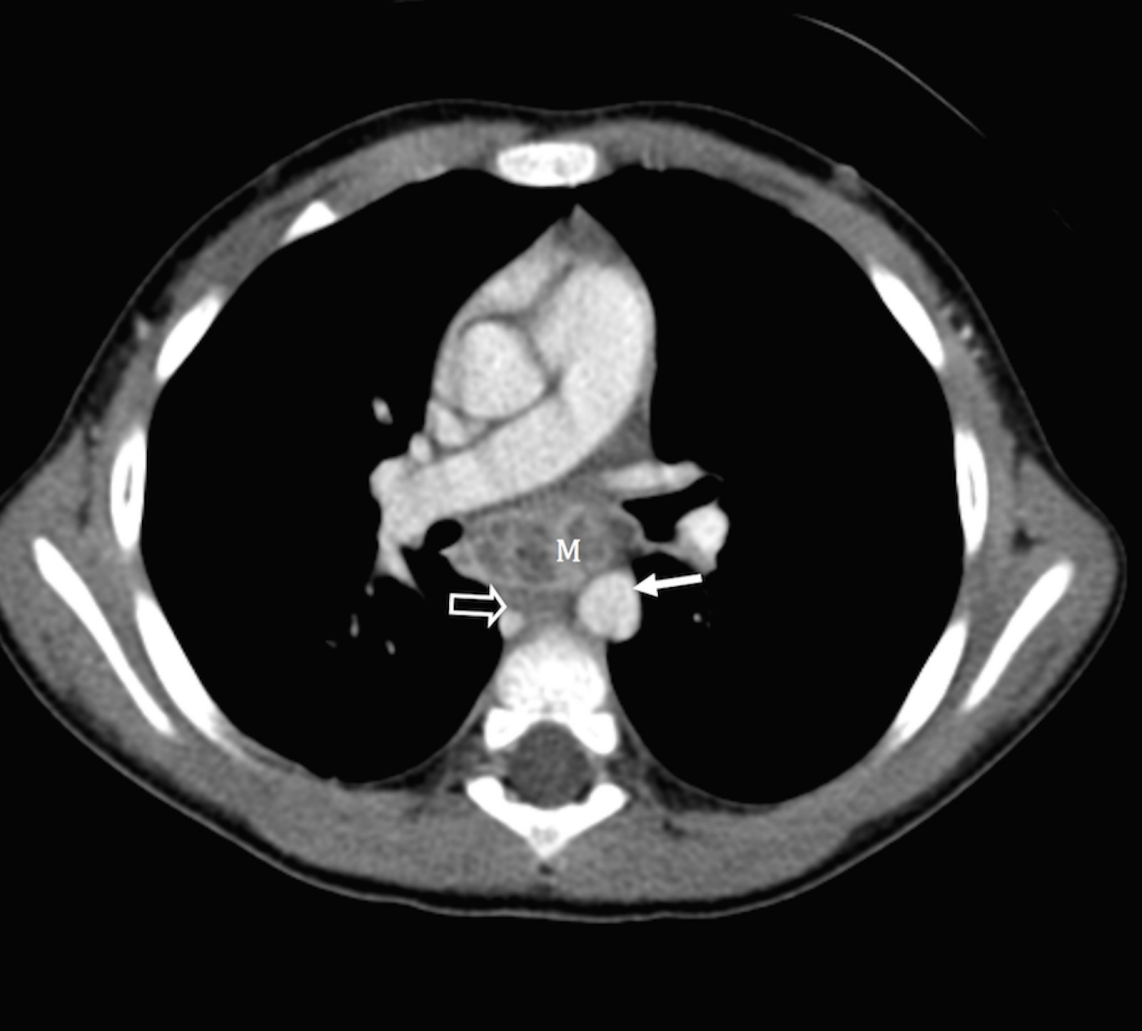
Axial post contrast CT of mediastinal mass Axial post contrast CT image in mediastinal window at subcarinal level shows a predominantly cystic peripherally enhancing subcarinal mass (M) abutting the descending thoracic aorta (arrow) and causing mass effect on the esophagus (open arrow).

**Figure 2:**
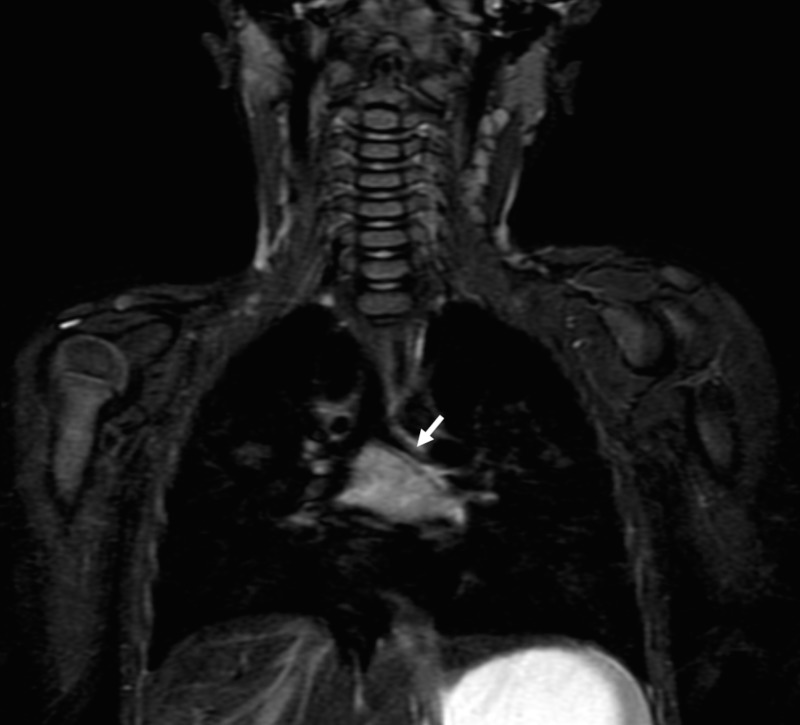
Coronal non-contrast T2 MRI image of mediastinal mass Coronal non-contrast T2 STIR (Short Tau inversion recovery) image shows a heterogeneous, mixed solid and cystic mass in subcarinal location causing elongation and at least moderate narrowing of the left main bronchus (arrow).

Rigid bronchoscopy revealed a large, non-ulcerated, occlusive mass originating from the inferior wall of the left mainstem bronchus. CT (Figure 1) and MRI (Figure 2) demonstrated a heterogeneous, mixed solid and cystic, peripherally enhancing subcarinal mass. Radiological appearance was not characteristic of any particular etiology.  Radiologic differential diagnosis was varied and included mixed venolymphatic malformation, teratoma, and other solid neoplastic lesions with necrosis as primary differentials.  Atypical infection was considered as less likely based on the radiological appearance of the mass. 

After consultation with the pediatric general surgery team, the patient underwent VATS with resection of the mediastinal mass. Intraoperatively, the mass was found to consist of white nodules filled with yellow fluid. Final pathology was consistent with necrotizing granulomatous infection with positive acid fast bacilli (AFB) stains for mycobacterium and DNA probe consistent with Mycobacterium avium. 

The patient improved following surgical treatment, and did not require any further medical treatment. Upon one-month follow-up after discharge from the hospital, he was doing well with no respiratory symptoms. 

## Discussion

This case highlights an uncommon presentation of pediatric atypical mycobacterium infection presenting with concern for foreign body and left main stem mass. Rates of MAC appear to be increasing in prevalence and severity since the 1970s in developed countries [1]. An array of factors has been cited as contributors including increased recognition of cases in immunocompetent hosts, environmental exposure, and enhanced virulence of infecting organisms [2].  A retrospective study analyzed cases of NTM in children and found rates in 2000-2004 almost doubled compared to 1990-1999 [1]. An increased severity of presentation has also been noted, with more cases of invasive pulmonary disease in the past 40 years [1]. Despite these trends, guidelines about the treatment of children with intrathoracic MAC infection remain unclear.

Cases of intrathoracic MAC infection in children are likely underdiagnosed and underreported. The presentation is often insidious in nature, with months of reported wheezing and shortness of breath.  This is often incorrectly treated as reactive airway disease, or foreign body ingestion as described in the present case study [2-3,6]. In other cases, children present with a more common initial symptom of cervical lymphadenitis [5]. This often prompts further diagnostic studies such as a chest x-ray that ultimately demonstrates a mass. Further conflicting this picture, children with MAC infection may have negative PPD and negative culture stains despite active disease [2].

Once intrathoracic MAC is diagnosed, there are varying options for successful treatment [2-4,6]. Piedimonte et al., described successful treatment of an immunocompetent child with MAC mediastinal lymphadenopathy using a combination of antimycobacterial drugs and laser bronchoscopy [6]. However, antimycobacterial agents have large morbidity profiles and are often not well tolerated. Several case studies have advocated surgical resection without antibiotics, with positive results [2-4]. Our case study describes successful treatment of an immunocompetent child with surgical excision of mediastinal MAC infection mass via VATS.

## Conclusions

The most common presentation of pediatric atypical mycobacterium infection in an immunocompetent child is cervical lymphadenopathy. However, increasing rates of MAC infection with intrathoracic disease have been described in the last several decades [1]. Children may present with a long-standing history of wheezing or shortness of breath, and are often misdiagnosed with reactive airway disease or airway foreign body. Physicians must keep intrathoracic MAC infection in their differential when treating patients with respiratory symptoms. Children with intrathoracic MAC have been successfully treated with surgical excision without antimycobacterial therapy. 
